# Telemedicine Examination of the Knee

**DOI:** 10.7759/cureus.37009

**Published:** 2023-04-01

**Authors:** Rock P Vomer, Emma York, Larick S Rayghan, Tarang Jethwa, Daniel P Montero, Christine Q Nguyen, George G. A Pujalte

**Affiliations:** 1 Department of Family and Community Health & Orthopedics, Division of Sports Medicine, Duke University, Durham, USA; 2 Family Medicine & Research, Mayo Clinic Jacksonville Campus, Jacksonville, USA; 3 Family Medicine, Eastern Virginia Medical School, Norfolk, USA; 4 Family and Community Medicine, Eastern Virginia Medical School, Norfolk, USA; 5 Family Medicine, Mayo Clinic, Jacksonville, USA; 6 Orthopedic Surgery, Mayo Clinic, Jacksonville, USA; 7 Family Medicine, Orthopedics, and Sports Medicine, Mayo Clinic, Jacksonville, USA

**Keywords:** gait, functional testing, virtual examination, telehealth, knee

## Abstract

Introduction

The coronavirus disease 2019 (COVID-19) pandemic has resulted in rapid healthcare system adaptations, including the acceptance of telemedicine in primary care. In the case of knee ailments, among the most common problems encountered in primary care, telemedicine provides a literal window to observe the patient performing functional activities. Despite its potential, there is a lack of standardized protocols for data collection. The purpose of this article is to provide a step-by-step protocol to aid in performing a telemedicine examination of the knee.

Methods

This article provides a step-by-step guide for a telehealth examination of the knee.

Results

A step-by-step examination of how to structure a telemedicine evaluation of the knee. A glossary of images of each maneuver has been included to demonstrate the components of the examination. Additionally, a table of questions and possible answers were included to help guide the provider through a knee examination.

Conclusion

This article provides a structured and efficient means of extracting clinically relevant information during telemedicine examinations of the knee.

## Introduction

In the setting of lockdowns and social distancing amidst a global pandemic, the benefits of telemedicine have become apparent. Throughout 2020, telemedicine has been rapidly accepted and incorporated into clinical practice to provide efficient and effective care. Telemedicine visits have been shown to be a viable mode of providing care for chronic disease, coordinating health maintenance, and evaluating musculoskeletal ailments [1]. Despite these benefits, a telemedicine evaluation poses challenges to both the patient and provider, as nonverbal signs of pain and change in function cannot be observed in the same manner as they would in person. These subtle clues help an experienced clinician in differentiating between common and rare pathologies. While patient history can still be collected verbally, the physical examination is done by the patient [1]. Though patient responses may lack details related to joint movement or tactile feedback from a special test, they are still highly valuable, as they often focus on the patient’s perceived functional deficit. With some creative thinking and patient partnership, it is still possible to conduct a thorough examination and provide effective care through telemedicine services.

The knee is among the most injured joints in the body; as a result, knee complaints are frequently encountered in the primary care setting. Primary care physicians are likely to encounter knee complaints with various causes that differ between adolescents and adults. Typical injuries are categorized as sprain or strain, contusion, meniscal, or ligamentous injuries [2]. Common etiologies include overuse, chronic disease, and acute traumatic injuries that require immediate attention. The knee is a large weight-bearing joint that includes three articulating surfaces, which form two distinct joints: patellofemoral and tibiofemoral. With any examination, patient history is vital and allows the clinician to develop a differential diagnosis that is either supported or refuted based on physical examination and diagnostic studies [2].

Traditionally, a tailored physical examination to evaluate a knee complaint involves inspection, palpation, range-of-motion (ROM) assessment, strength testing, sensation assessment, and special testing. Telemedicine examinations have the limitation of not being able to physically assess the patients' strength and perform the traditional special tests as would be done in an in-person visit. Despite this, telemedicine encounters for knee examinations provide additional opportunities and benefits to patients and providers alike. For patients, it allows for expanded access, reduces wait time for services, limits travel, and is cost-efficient [3]. For the provider, it allows for efficient triage, monitoring, and improved allocation of medical resources. Telemedicine enables the provider to not only evaluate how the patient moves in their home environment but also to address potential fall risks and assess the need for assistive devices to improve the functional independence of the patient [3]. 

As the use of telemedicine for patient encounters increases, protocols should be developed to provide direction on how to efficiently evaluate a knee complaint. Recently, the American Medical Association acknowledged the need for telemedicine training and encouraged its incorporation into medical education programs [4]. The Liaison Committee on Medical Education’s Annual Medical School Questionnaire from 2015 to 2016 observed that over 25% of the nation’s allopathic medical schools have added telemedicine training in the preclinical years of their curriculum and about half have implemented it in clerkship [4]. As medical schools continue to incorporate telemedicine education, these protocols could be beneficial resources for future medical students. Likewise, protocols can be used in continuing medical education as time-efficient updates for practicing clinicians. This paper outlines possible questions, responses, and video examination techniques to aid the clinician in conducting an effective telemedicine knee examination.

## Materials and methods

Inspection

Have the patient stand with feet shoulder-width apart, preferably in fitted athletic garments. Postural assessment should include anterior, lateral, and posterior views. When assessing knee complaints, the evaluation should include the entire kinetic chain from the lumbar spine to the foot. Inspect for asymmetry in lumbar lordosis, anterior superior iliac spine, iliac crest height, and rotation. Then, examine the knee from an anterior, lateral, and posterior view to assess the position of the patella and fibular head. Estimate the Q angle of the knee in the anterior and posterior views. If the angle is excessively increased or decreased, it can play a role in force absorption on the lower kinetic chain and can be a source of pain at the hip. Next, ask the patient to point to the area of maximal pain and draw the radiation pattern with their finger. Finally, proceed to inspect the ankle and hindfoot to identify pronation or supination of the foot and tibia-to-floor angle. Tibia-to-floor angles greater than 10° can alter the mechanics of the lower extremity and contribute to knee pain [5].

Palpation

Have the patient palpate the quadriceps tendon, iliotibial band, fibular head, patella, medial and lateral joint surfaces, and the joint line to assess pain. Ask the patient to comment on the muscle mass, temperature, and sensation of the anterior, medial, and lateral aspects of the thigh and leg [6]. As the patient palpates the knee joint, inquire about the quality and shape of the joint. Next, direct the patient to palpate the popliteal fossa to assess for cysts or masses. When the patient is palpating the patella, instruct them to perform ballottement testing [7]. Lastly, have the patient assess for distal pulses and tortuous venous congestion.

ROM assessment

ROM testing is a valuable component of a thorough knee examination. As with any joint examination, it should compare the affected to the unaffected side. ROM testing should appreciate active ROM and tissue end-feel (Table 1). All ROM testing will be approximated via telemedicine, with an emphasis on comparison with the unaffected limb.

**Table 1:** Knee range of motion and possible end-feels

Motion	Range	End-Feel
Flexion	0°-140°	Soft tissue approximation
Extension	0°-15°	Soft tissue stretch
External tibial rotation	30°-40°	Soft tissue stretch
Internal tibial rotation	20°-30°	Soft tissue stretch

First, instruct the patient to sit in a chair with hips flexed to 90°. Move the ankle inward and outward, assessing external and internal ROM of the tibia while monitoring for pain. In this same position, ask the patient to flex the knee past 90° and assess for pain. During flexion, it is also important to determine when patellar pain occurs. Have the patient keep a record of when the pain starts. Knee extension can be assessed in a seated or standing position, though a seated position is ideal for both patient comfort and to keep the examination sequential.

Strength testing, functional assessment, and gait analysis

Strength testing via telemedicine is possible by replacing manual muscle testing with functional assessments. For general lower body strength testing, have the patient perform a standard body squat. This motion should be assessed from both the lateral and posterior views. If the patient can perform this against gravity, their strength level is at least 3/5. On posterior assessment, hip shifting can be assessed. If the patient shifts away from a hip, this is a positive hip shift test, indicating that there is decreased mobility or strength in that hip. This weakness or restricted mobility can be a contributing factor to knee pain. Next, establish a baseline by having the patient perform a single-leg squat with the unaffected limb. Repeat the single-leg squat with the affected limb to assess for hip weakness, dynamic genu valgus, and pain. Then, have the patient perform toe raises: first with both limbs, then individually beginning with the unaffected limb. It is important to note the contraction pattern of the gastrocnemius muscle in both the concentric and eccentric phases. This motion should be observed from the posterior view of the patient.

A gait assessment is also valuable in a thorough knee evaluation. Possible gait abnormalities that can be seen via telemedicine include the Trendelenburg, foot slap, and antalgic gait patterns. During the gait examination, assess for stance width, stance time, and stride length.

Special testing

Special testing for knee complaints via telemedicine should involve both seated and standing tests. Sequential testing will likely make the examination easier for the patient. First, have the patient continue standing after examining for gait pattern. Then, instruct the patient to supply medial and lateral gapping forces to evaluate the medial and lateral collateral ligaments. Next, have the patient perform Thessaly testing to assess the meniscus. If the patient is unable to balance on one leg, they can be assisted by another individual or use a stable surface for support. During this test, it is important to observe the effect of axial compression and torsional forces on the meniscus. Direct the patient to take a seated position, giving you a lateral view as they perform patellar and iliotibial band testing.

## Results

History

When a patient presents with any musculoskeletal problem, it is important to first illicit a thorough history of the presenting complaint. Pertinent elements of the history include the problem onset and the position, quality, severity, and radiation of the pain. It is important to understand the time course of the problem (i.e. has the pain changed over time). It is important to understand what aggravates the patient's pain as well as any treatments the patient has already tried to alleviate the pain. Asking about associated symptoms, such as sensory, skin, and temperature changes, as well as systemic symptoms, such as fever/chills, will aid in triage to further evaluation.

Physical exam

Next, the provider and patient will perform an interactive telehealth physical exam. Clear and direct communication is key to the success of this part of the virtual encounter. Table 2 outlines a step-wise series of questions to ask the patient with implications and considerations for each response [8-19].

**Table 2:** Knee evaluation by telephone: questions/instructions and considerations Abbreviations: ITB, iliotibial band; MCL, medial collateral ligament; OA, osteoarthritis; PCL, posterior cruciate ligament; PFJ, patellofemoral joint

What to ask	Possible responses	Implications of responses
Inspection		
Do you notice any sunken, swollen, bruised, or red areas?	Affirmative response	Sunken areas, possible areas of atrophy; swollen, bruised, or red areas, possible injury ecchymosis.
Do you notice any frank deformity of your affected knee compared to the other side?	Affirmative response	Possible fracture.
When looking from the side, do you notice one knee to be more extended compared to the other?	Affirmative response	Possible signs of general ligamentous laxity, patella alta hyperextension injury, or posterior cruciate ligament (PCL) sprain [8].
Do you notice that one leg appears longer than the other?	Affirmative response	Possible excessive pronation, toeing-out, or other alteration in the gait cycle, leading to increased compressive forces during gait; possible rotated innominate, producing a physiologic short leg [10].
When looking from the front, do you notice that your knees bow in or out? (Figure 1)	Affirmative response	Possible genu varus (“bow-leggedness”) or genu valgus (“knock-kneed”) causes a shift of compressive forces at the knee, potentially leading to increased degeneration in either the medial or lateral joint compartments in the knee; genu valgus could predispose to lateral patellofemoral pain syndrome, chondromalacia, or chondral injury [6].
Do you notice increased knee bending when you stand? Do you notice knee joint squaring when you sit? (Figure 2)	Affirmative response	Possible osteoarthritis (OA).
From a seated position, does one shin look more internally rotated than the other?	Affirmative response	Possible internal rotation of the tibia leading to excessive or altered loading forces on the knee; possible somatic dysfunction of the tibia.
From a posterior view, are your feet flat? (Figure 3)	Affirmative response	“Too many toes” sign suggests the source of pain may be hyperpronation of the foot causing altered mechanics and force distribution.
Palpation		
With your leg straight, place one hand above your kneecap, and with the other hand, tap the knee. Do you notice a floating sensation? (Figure 4)	Affirmative response	Positive for patellar ballottement; possible synovial joint effusion, hemarthrosis, medial plica syndrome, disruption of the joint capsule, or popliteal swelling [12].
When palpating the front of your knee, do you notice sunken, swollen, or tender areas or decreased muscle mass in the anterior thigh muscles?	Affirmative response	Swollen areas, possible synovial effusion or ecchymosis; tender areas, possible tendinitis or tendinosis; exquisite pain and loss of muscle mass, possible rupture of the quadriceps tendon.
Palpate directly above and below your kneecap. Do you notice any pain in either spot?	Affirmative response	If pain is appreciated distal to the patella, possible patella tendon-ligament injury.
When you palpate the inside of your knee do you notice pain?	Affirmative response	Possible medial collateral injury, pes anserine bursitis, or OA.
When you palpate the outside of your knee do you notice pain? (Figure 5)	Affirmative response	Possible patellofemoral joint (PFJ) pain, iliotibial band (ITB) syndrome, somatic dysfunction of the fibular head, or peroneal nerve compression.
When you palpate the joint line between the thigh and shin bones, do you have pain? (Figure 1)	Affirmative response	Possible OA, patellar tendon tendonitis, or PFJ involvement [18].
Range of motion		
From a seated position, can you fully extend your knee? (Figure 2)	Negative response	Possible tightness/restriction in the hamstring group or muscle inhibition/guarding.
When you straighten your knee, does the kneecap follow a C or J shape pattern? (Figure 3)	C shape or J shape	If J shape pattern (“J sign”), possible patellar instability.
From a seated position, can you fully flex your knee? (Figure 2)	Negative response	Possible distal patellar lesions if limited in early flexion; possible proximal patellar lesion if limited at 90° of flexion; possible OA or free joint body with a hard stop during knee flexion [3].
From a seated position, can you fully internally rotate your shin?	Negative response	Possible somatic dysfunction of the tibia.
From a seated position, can you fully externally rotate your shin?	Negative response	Possible somatic dysfunction of the tibia.
Functional and gait assessment		
Do you notice your foot “slapping” when you walk?	Affirmative response	Possible peroneal nerve entrapment at the fibular head or tibiofibular syndesmosis [15].
Does your hip drop when you walk? (Figure 4)	Affirmative response	Positive Trendelenburg sign; possible weakness in the glute medius of the affected limb.
Do you have pain when you walk?	Affirmative response	Antalgic gait pattern.
Is the injured knee more flexed than the other when you walk?	Affirmative response	Possible quadriceps-avoidance gait, a compensation pattern to reduce the anterior translation of the tibia by shifting active knee stabilization to the hamstring group, suggesting anterior cruciate ligament injury [7].
Do you have increased knee pain when you go up and down stairs?	Affirmative response	Possible PFJ involvement or patellar chondromalacia [16].
When you perform a bodyweight squat, do you notice pain, anterior movement of your knee, the medial collapse of your knee, or hip shifting? (Figure 5)	Affirmative response	Pain, possible PFJ involvement; excessive anterior translation of the knee, possible laxity; medial collapse and pain, possible medial collateral ligament (MCL) involvement and weakness in involved extremity; hip shifting, possible weakness or restriction contributing to pain in side shifted away from.
Stand and balance on your left/right leg. Slowly lower yourself into a single-leg squat. Does this cause pain? Do you feel unstable?	Affirmative response	Possible hip stabilizer weakness, patellofemoral pain syndrome, or lumbar radiculopathy.
Strength testing		
Against gravity, can you fully extend your knee?	Affirmative response	The quadriceps group has at least 3/5 strength [1].
Against gravity, can you fully flex your knee?	Affirmative response	The hamstring group has at least 3/5 strength [1].
Against gravity, can you perform a calf raise?	Affirmative response	Plantar flexors have at least 3/5 strength [1].
Laying on your side, can you raise your top leg up from your bottom leg?	Affirmative response	The hip abductor group has at least 3/5 strength [1].
Laying on your back, can you perform a bridge with both legs and with one leg?	Affirmative response	The hip extensor group has at least 3/5 strength [1].
Special testing		
From a standing position, can you bend your knee in? Does this produce pain? (Figure 6)	Affirmative response	Positive modified valgus test; possible MCL sprain or tear or damage to the posteromedial capsule or posterior oblique ligament [1].
From a standing position, can you bend your knee out? Does this produce pain? (Figure 6)	Affirmative response	Positive modified valgus test, possible lateral collateral ligament, lateral capsular ligament, or arcuate-popliteus complex injury.
Hold your thumb over the top of the outside of your knee. Now straighten the knee. Does this produce pain? (Figure 7)	Affirmative response	Positive modified Noble compression test, possible ITB syndrome or fibular head dysfunction [13].
Lay on your back and flex your hip and knees to 90 degrees. Place your ankles on a small support. Do you notice a positive drop in one of the knees? (Figure 8)	Affirmative response	Positive modified posterior sag sign, suggesting abnormal tibial translation; possible disruption or injury to PCL [1].
With your leg straight, are you able to lift the outside of your kneecap up from the femur?	Affirmative response	Positive patellar tilt test, suggesting tight lateral retinacula structures or ITBs; possible chondromalacia or chondral pathology.
Please extend your knee, and now provide over pressure with your hands. Does his produce pain?	Affirmative response	Positive patellar compression test, suggesting PFJ degeneration.
Sit at the edge of the bed. Pull one knee toward your chest and lay flat on your back. Do you have pain in the hip that is straight? Do you notice hip flexion, knee extension, rotation of your lower leg, or outward rotation of your hip? (Figure 9)	Affirmative response	Positive modified Thomas test; if the knee is extended, possible rectus femoris involvement; if the hip is flexed, possible psoas involvement; if lower leg rotation, possible ITB restrictions or ITB syndrome; if abduction of the hip, possible tensor fascia latae involvement [17].
Stand on one leg with your knee slightly bent. Rotate your trunk to the left and right. Does either motion produce pain? (Figure 10)	Affirmative response	Positive modified Thessaly test; possible meniscal pathology.
Special considerations		
Do you notice a decrease or increase in sensation around your knee?	Affirmative response	Possible compression of the peroneal or tibial nerve.
Do you feel a grinding sensation in your knee?	Affirmative response	Possible OA, meniscal degeneration, or free body in joint space.
Does your knee feel like it is going to give way?	Affirmative response	Possible ligamentous or meniscal injury.
Have you experienced a dashboard injury?	Affirmative response	Possible PCL sprain or tear.
Do you have sensation and intact pulses in the foot or calf of the affected leg?	Negative response	If diminished pulses, possible injury to the popliteal artery; if diminished sensation, possible tibial or peroneal nerve involvement.
Is the patient an adolescent with knee pain without a specific mechanism of injury?	Affirmative response	Possible slipped capital femoral epiphysis or viral tenosynovitis.
Do you have any abrasion, recent laceration, recent surgery, or increased warmth, swelling, or erythema of the knee? Do you have systemic signs of illness?	Affirmative response	Possible septic arthritis, gouty arthritis, pseudo gout, or cellulitis.
Have you experienced significant trauma, such as a fall or blunt force injury?	Affirmative response	Possible fracture.

**Figure 1:**
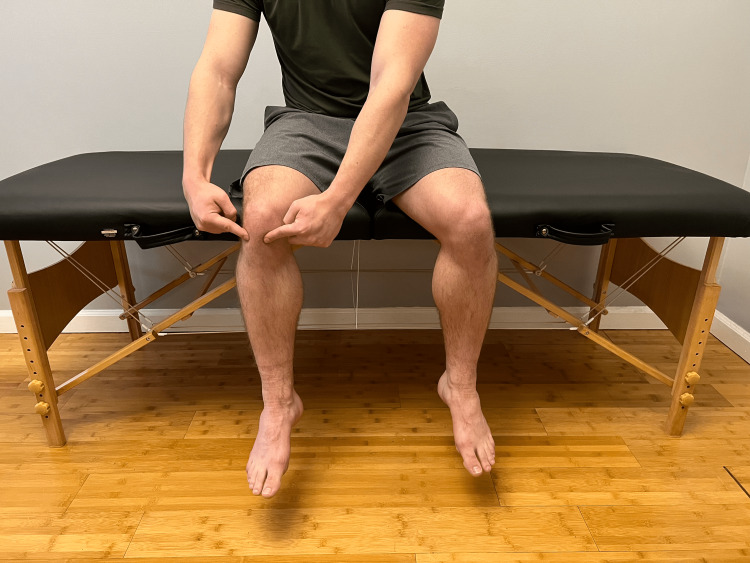
Joint line of the knee Palpation of the joint line that reproduces pain suggests possible osteoarthritis, patellar tendinitis, or proximal tibiofibular joint injury.

**Figure 2:**
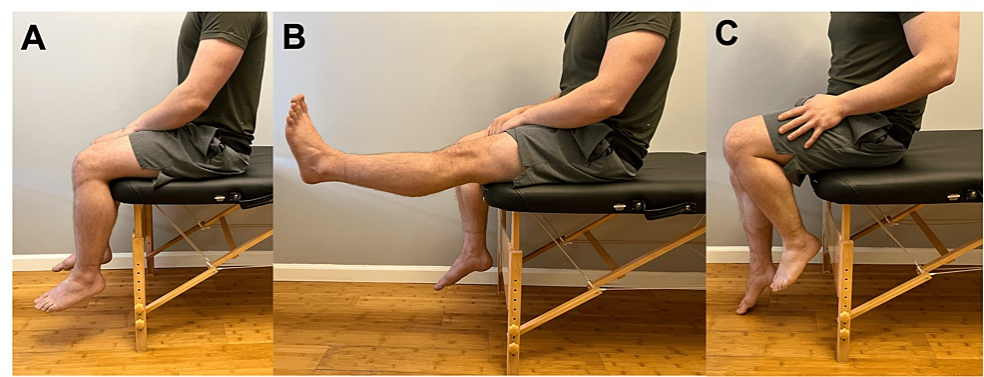
Active knee flexion and extension A. Lateral view of the patient for knee range of motion approximation. B. There should be 0° to 15° of knee extension; limited extension suggests hamstring restriction. C. There should be approximately 120° to 140° of knee flexion; limited flexion can be caused by quadriceps or patellar pathology.

**Figure 3:**
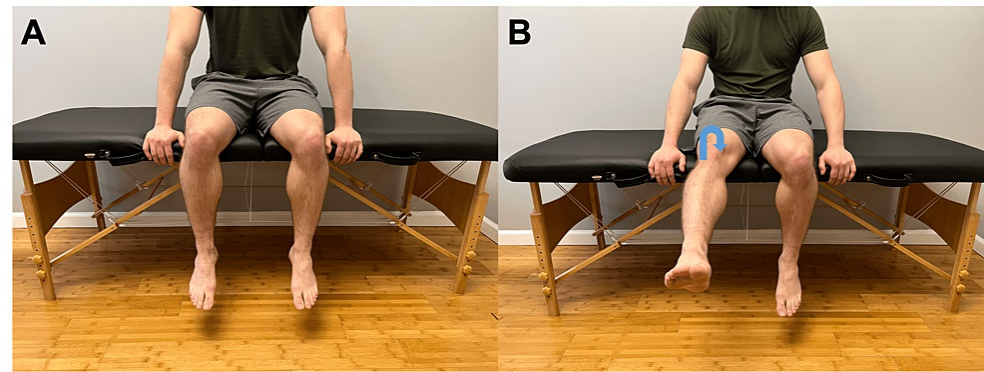
J sign A. During knee extension, the patella should follow a smooth arc. B. A J-shaped pattern (J sign) suggests patellar instability.

**Figure 4:**
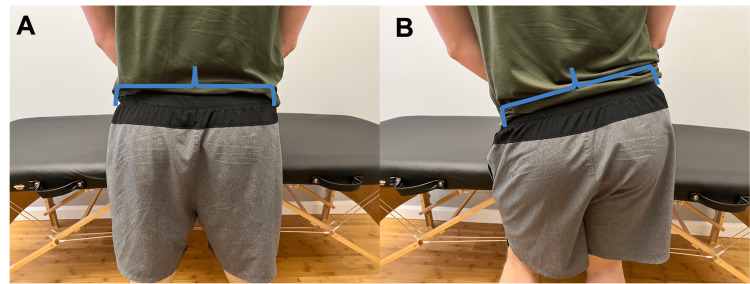
Trendelenburg gait A. During normal gait, the hip abductors stabilize the pelvis and keep it level. B. When there is hip abductor weakness, the hip is not level during the gait cycle. This lack of stability can alter force distributions in the kinetic chain and contribute to knee pain.

**Figure 5:**
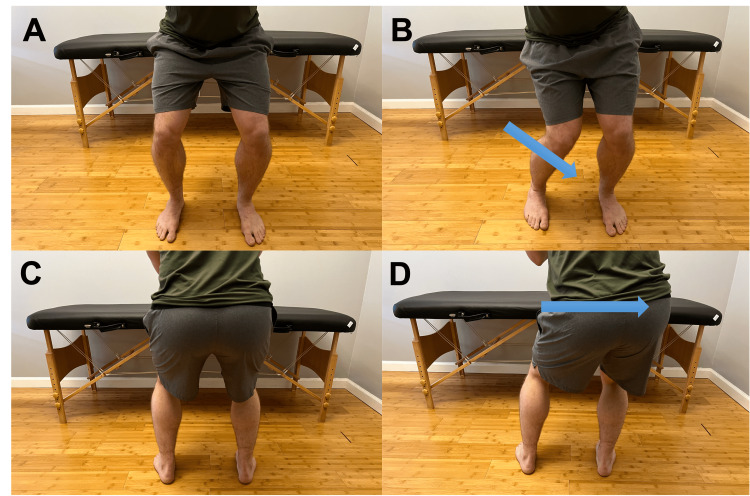
Body weight squat A. Anterior view of smooth controlled squat descent. B. Medial collapse of the knee during descent (arrow) with pain suggests medial collateral ligament involvement and weakness in the involved extremity. C. Posterior view of smooth controlled squat descent. D. Hip shift (arrow) suggests weakness or restriction of motion in the side shifted away from.

**Figure 6:**
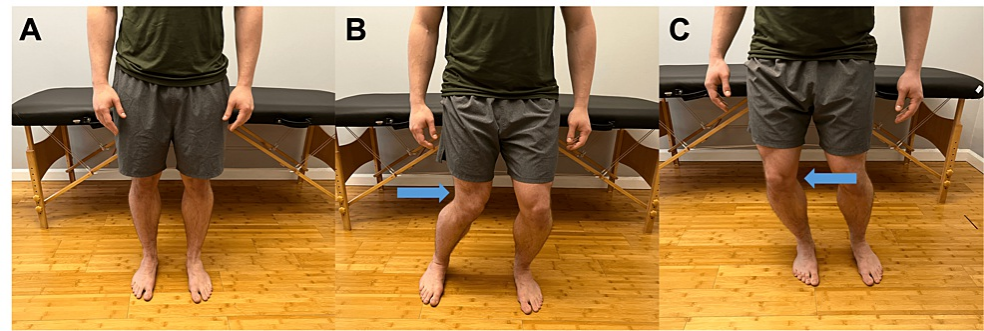
Modified medial and valgus stress testing A. Neutral standing position. B. Modified valgus stress. Instruct the patient to bend their knee medially (arrow). C. Modified varus stress. Instruct the patient to bend their knee laterally (arrow). Pain with either maneuver suggests ligamentous involvement on the side of the knee that is stressed.

**Figure 7:**
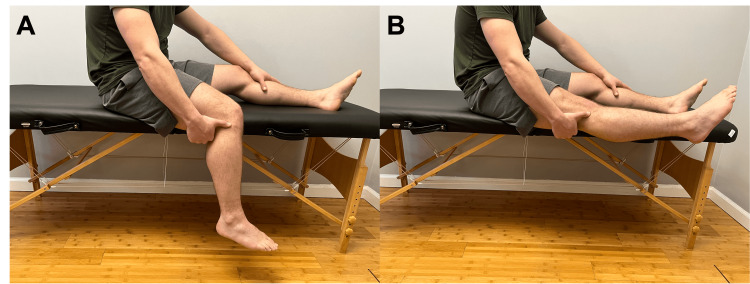
Modified Noble's compression test Instruct the patient to apply pressure at the distal insertion iliotibial band (A) and then extend the knee (B). Pain suggests possible iliotibial band syndrome or fibular head dysfunction.

**Figure 8:**
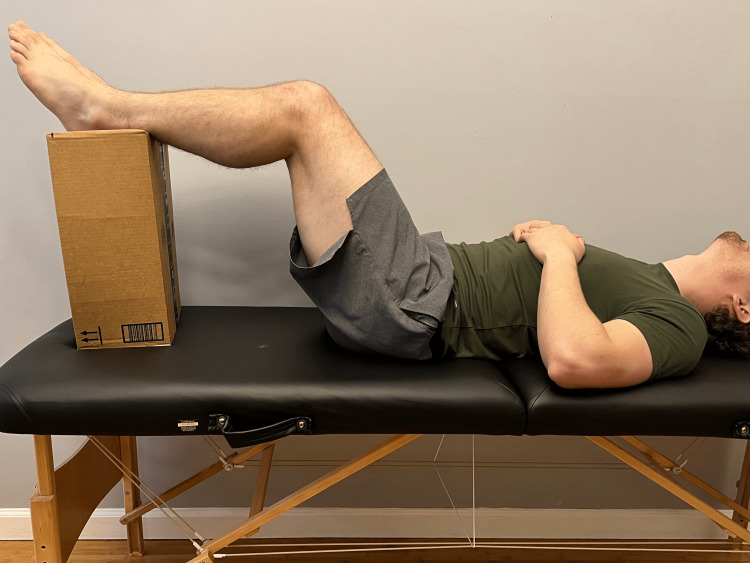
Posterior sag sign Instruct the patient to lay supine with knees at approximately 90° flexion, supporting the heels with a box. A noticeable difference in tibial height represents a posterior sag sign, suggesting the involvement of the posterior cruciate ligament of the knee.

**Figure 9:**
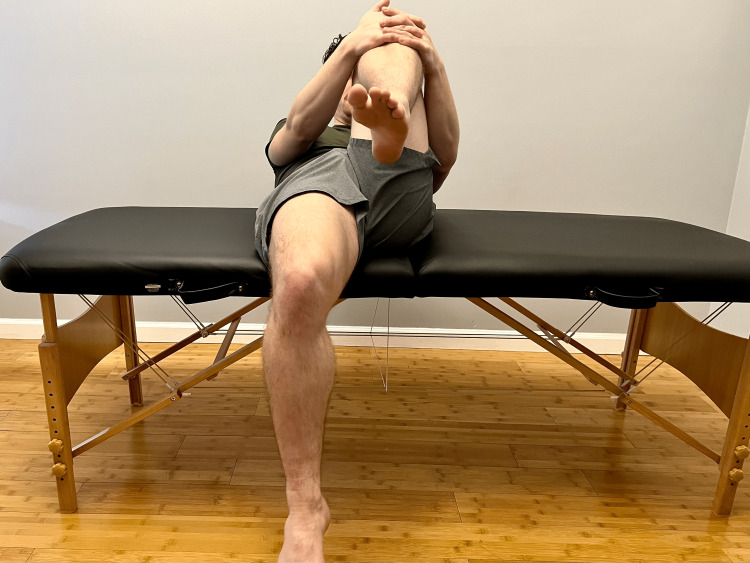
Modified Thomas test Instruct the patient to pull the unaffected knee to the chest and lay back. If the knee is extended, there is possible rectus femoris involvement. If the hip is flexed, there is possible psoas involvement. Rotation suggests iliotibial band restrictions or iliotibial band syndrome. Abduction of the hip suggests possible tensor fascia latae involvement as the source of pain.

**Figure 10:**
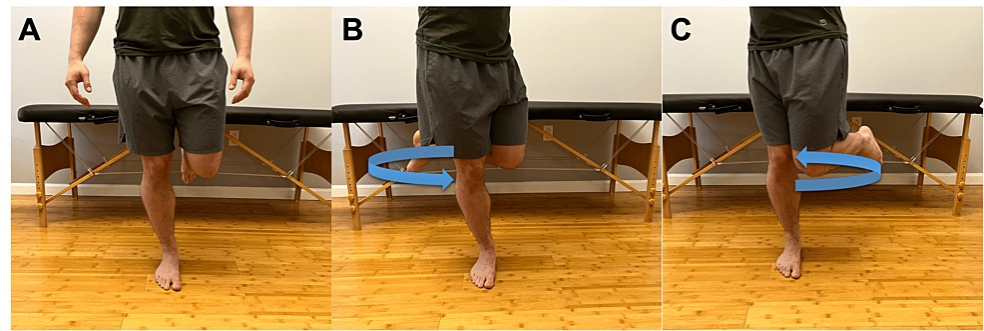
Modified Thessaly test A. Instruct the patient to stand on the affected leg with slight knee flexion. Have the patient turn their shoulders to the left (B) and right (C). Knee pain with this maneuver suggests possible meniscal pathology.

Inspection, Palpation, Range of Motion, and Strength Testing

The telehealth physical exam begins with an inspection in which the provider asks the patient about skin changes, obvious deformities, and other subtle anatomic differences that can be appreciated in specific positions. Next, the provider will walk the patient through palpation of the knee, eliciting information regarding pain, tissue feel, sensation, and swelling (Figure 1). Next, the provider will assess active ROM (Figure 2). The provider's shoulder also assesses the quality of patellar tracking and the presence of a J sign (Figure 3). The provider may also instruct the patient to perform passive ROM by asking them to fully relax their leg and use their hands to move the knee through flexion and extension. Table 1 outlines standard ROM measurements and expected end-feel for knee flexion/extension and tibial internal/external rotation. The provider will then perform a strength test to assess the quadriceps, hamstring, plantar flexor, hip abductor, and hip extensor muscle groups against gravity. Of note, using this method, the provider can only classify strength as "at least 3/5," as there is no resistance force applied. The provider can inquire about subjective weakness to understand potential strength deficits.

Functional Assessment and Gait Analysis

The provider next performs a functional assessment and gait analysis. The provider should inquire about abnormal movement patterns with walking such as foot and hip drops. The patient will be asked to walk toward and away from the camera to appreciate any gait abnormalities (Figure 4). The provider will additionally inquire about pain and instability during specific functional movements such as stair climbing or descending and both single and double-leg squatting (Figure 5). The implications of each of these findings are referenced in Table 2.

Special Tests

The final element of the telehealth knee physical exam includes a series of modified special tests. These include both varus and valgus testing, modified Noble's compression test, posterior sag sign, patellar tilt test, patellar compression test, modified Thomas test, and modified Thessaly test (Figures 6-10) [1,9,11,13,14,17]. These special tests and their clinical implications are outlined in Table 2.

Diagnosis, treatment, and further evaluation

Synthesizing the history and all components of the telehealth physical exam, the provider should be able to effectively diagnose and categorize the knee complaint as an emergent or non-emergent situation. In the event of an acute situation, the provider can direct the patient to the appropriate type of acute care setting. In the event of a non-emergent problem, the provider can innate conservative care plans and arrange for patient follow-up.

## Discussion

While telemedicine has been used by clinicians for many years, its utility and importance became increasingly evident during the COVID-19 pandemic. Initially, telemedicine was intended to expand access to care for patients in remote areas, but its scope has broadened and is now a common appointment type offered by a variety of medical specialists. Telemedicine has been widely accepted by both patients and providers. One of the major advantages of telemedicine encounters is that patients can be rapidly triaged to the appropriate level of care. An example of this would be a provider identifying a patient with an acute injury like a fracture and directing them to an acute care setting. Patients consistently report a high satisfaction rate with telemedicine appointments and tend to cite the convenience of decreased travel times and costs as the main drivers for this satisfaction [19]. In general, providers also report high satisfaction rates as long as there is administrative support, reliable technology, and adequate reimbursement [19].

Telemedicine examinations are a new and rapidly developing form of patient encounter that has limitations that providers should acknowledge. The first limitation of telemedicine examinations is that to conduct this type of examination, the patient needs to have access and the ability to utilize an Internet-connected device [7]. The second limitation is that telemedicine examinations require a reliable internet connection for the patient and provider to communicate. The telehealth examination itself does not allow for traditional palpation and strength and special testing and relies on patient-performed testing to obtain information [9]. This limitation can be overcome with structured examination protocols, visual aids for the patient, and provider training in telemedicine.

Given that musculoskeletal conditions are among the primary reasons individuals seek medical care, it is important to have a standardized framework for the musculoskeletal exam to ensure proper diagnosis and treatment [3,20]. A recent review concluded that telemedicine services achieve an average agreement of 62% with in-person knee assessments, highlighting the importance of standardizing the virtual approach and training providers to adapt their typical physical exam in a virtual environment [21]. The virtual musculoskeletal knee examination presented in this article provides a reliable and easy-to-follow, step-by-step guide with clinical implications of potential findings. While telemedicine can never replace the in-person knee physical exam, using this framework, providers can expedite patient care through proper triage for further evaluation with imaging and directing the next steps in treatment as appropriate. A low threshold for knee imaging should be maintained in the following situations: inability to bear weight, severely antalgic gait, knee swelling, erythema, trauma to the hip, night-time pain, or fever/chills with pain. Immediate referral to emergency or in-person evaluation is warranted for direct blunt trauma, falls from an elevation, and otherwise medically frail patients.

## Conclusions

Thorough musculoskeletal examinations are important for patients with knee complaints. Structured examination protocols enable the effective evaluation of knee complaints via telemedicine. Providers can expedite care and effectively triage patients to the appropriate level of care. Telemedicine can also identify when imaging is warranted by identifying symptoms, including an inability to bear weight, severely antalgic gait, knee swelling, erythema, knee or hip trauma, night-time pain, or fever and chills with pain. Telemedicine encounters can be used to evaluate and treat knee pain when the patient is unable to drive or local access to a clinic is limited. Future investigations on cost-benefit analyses and how to incorporate video aids and measuring tools effectively have the potential to improve telemedicine care.
